# *Chlorella vulgaris* algae ameliorates chlorpyrifos toxicity in Nile tilapia with special reference to antioxidant enzymes and *Streptococcus agalactiae* infection

**DOI:** 10.1007/s11033-024-09535-0

**Published:** 2024-05-09

**Authors:** Walaa S. Tawfeek, Amina S. Kassab, Eman T. Al-Sokary, Mona E. Abass, Ahmed H. Sherif

**Affiliations:** 1 Fish Disease Department, Animal Health Research Institute AHRI, Agriculture Research Center ARC, Dokki, Kafrelsheikh, 12619 Egypt; 2 Biochemistry, Nutritional Deficiency Diseases and Toxicology Unit, Animal Health Research Institute AHRI, Agriculture Research Center ARC, Kafrelsheikh, 12619 Egypt

**Keywords:** Antioxidant enzymes, Chlorpyrifos, *Chlorella vulgaris*, Cytokines, *Oreochromis niloticus*, *Streptococcus agalactiae*

## Abstract

**Background:**

Chlorpyrifos (CPF) is a widely used pesticide in the production of plant crops. Despite rapid CPF biodegradation, fish were exposed to wastewater containing detectable residues. Recently, medicinal plants and algae were intensively used in aquaculture to replace antibiotics and ameliorate stress impacts.

**Methods and results:**

An indoor experiment was conducted to evaluate the deleterious impacts of CPF pollution on Nile tilapia health and the potential mitigation role of *Chlorella vulgaris* algae. Firstly, the median lethal concentration LC_50 − 72 h_ of CPF was determined to be 85.8 µg /L in Nile tilapia (35.6 ± 0.5 g body weight) at a water temperature of 27.5 °C. Secondly, fish were exposed to 10% of LC_50 − 72 h_ for six weeks, and tissue samples were collected and examined every two weeks. Also, Nile tilapia were experimentally infected with *Streptococcus agalactiae.* Exposed fish were immunosuppressed expressed with a decrease in gene expressions of interleukin *(IL) 1β, IL-10*, and tumor necrosis factor *(TNF)-α.* Also, a decline was recorded in glutathione peroxidase *(GPx)*, superoxide dismutase *(SOD)*, and catalase *(CAT)* gene expression in the head kidney tissue. A high mortality rate (MR) of 100% was recorded in fish exposed to CPF for six weeks and challenged with *S. agalactiae.* Fish that received dietary *C. vulgaris* could restore gene expression cytokines and antioxidants compared to the control. After six weeks of CPF exposure, fish suffered from anemia as red blood cell count (RBCs), hemoglobin (Hb), and packed cell volume (PCV) significantly declined along with downregulation of serum total protein (TP), globulin (GLO), and albumin (ALB). Liver enzymes were significantly upregulated in fish exposed to CPF pollution, alanine aminotransferase (ALT) (42.5, 53.3, and 61.7 IU/L) and aspartate aminotransferase (AST) (30.1, 31.2, and 22.8) after 2, 4, and 6 weeks, respectively. On *S. agalactiae* challenge, high MR was recorded in Nile tilapia exposed to CPF (G3) 60%, 60%, and 100% in week 2, week 4, and week 6, and *C. vulgaris* provided a relative protection level (RPL) of 0, 14.29, and 20%, respectively.

**Conclusions:**

It was concluded that CPF pollution induces immunosuppressed status, oxidative stress, and anemic signs in Nile tilapia. In contrast, *C. vulgaris* at a 50 g/kg fish feed dose could partially ameliorate such withdrawals, restoring normal physiological parameters.

## Introduction

According to the latest FAO report, Nile tilapia *(Oreochromis niloticus)* is one of the highest-farmed fish species globally; Egypt became among the highest producers, ranking 11th [1].

Pesticides are widely used in plant crop production, and they can reach water streams and aquatic environments [2], allowing increasing bioaccumulation in the tissues of different aquatic animals [3]. Pesticides enter aquatic environments through their extensive agricultural and domestic use, and approximately 64% of agricultural water sheds worldwide are at risk of pesticide pollution [4].

Chlorpyrifos (CPF) [O, O-diethyl-O-(3,5,6-trichloro-2-pyridyl) phosphorothioate] is considered one of the widely used organophosphate pesticides owing to the broad-spectrum of eradicating plant pests and eliminating mosquitoes, the released CPF contaminate the aquatic environment and remain for 8–53 days till decomposition [5]. CPF has highly absorbable properties via the gills, skin, and digestive system of aquatic animals; it bioaccumulates in their tissues (liver and kidney), and its residues have been discovered in farmed and wild fishes, hindering their normal metabolic functions and threatening their life, in addition to causing genotoxicity [6, 7].

Pesticide exposure suppresses fish immunity, adversely impacting cytokine gene expressions such as interleukin *(IL)-1β, IL-8*, and tumor necrosis factor *(TNF)-α* [8], making them vulnerable to infectious diseases [9]. Also, Pesticide bioaccumulation could generate reactive oxygen species (ROS), injuring different fish tissues, oxidative stress has recently been hypothesized to be the main mode of CPF toxicity. Antioxidant enzymes are released to detoxify generated ROS, such as glutathione-S transferase (GST), superoxide dismutase (SOD), catalase (CAT), and glutathione (GSH) to counteract oxidative damage [10]. The exposure of fish to pesticides is unavoidable, despite legal restrictions on the use of pesticides that can effectively reduce environmental contamination. Also, improving the biological and physiological status of the fish can ameliorate the toxic withdrawals of pesticides [11].

Pollution, antibiotics, and chemotherapy mainly affect the antioxidant status of aquatic animals. In the case of bacterial infection, the mortality rate, clinical signs, and treatment efficacy are impacted by the antioxidant-immune status of diseased fish. So, pollution and bacterial infection could act synergistically to increase the mortality rate and provoke more prominent clinical signs.

Some natural products could mitigate the impacts of several toxicants by increasing antioxidant capacity protecting fish tissues [12–19]. Supplementation with dietary lycopene, chlorella, or citric acid could fully or partially mitigate the impacts of environmental toxicants, which could enhance the antioxidant capacity in African sharp-tooth catfish *(Clarias gariepinus)* [20].

*Chlorella vulgaris* is a freshwater green algae that contains different components: 60% protein and 18 amino acids, fiber, vitamins, and minerals, in addition to bioactive substances such as antioxidants and chlorophylls. Recently, *C. vulgaris became* one of the most frequently used microalgae in aquatic animal diet formulation. Many studies have assessed the ability of *C. vulgaris* to improve growth, immune responses, and stress amelioration. Also, it combats disease resistance in fish by inhibiting bacterial quorum sensing [21, 22]. It was used as a feed additive for aquatic organisms at a rate of 2.5%, 0.5%, 7%, and 10% of fish feed [23, 24]. Dietary C. *vulgaris* was used in fish to alleviate the adverse impact of exposure to microplastic [25] and CPF [26], in addition to maintaining the growth performance and biochemical parameters. Inconsistent [27], chlorella algae effectively ameliorates the depressed responses of innate immune and oxidative stress caused by arsenic contamination, suggesting a potential therapeutic role. The antioxidant property of *C. vulgaris* can counteract sodium nitrite-induced toxicity and prevent oxidative stress [28].

This work is a trial to mitigate Nile tilapia’s immunosuppression and oxidative stress caused by CPF exposure. To counteract S*treptococcus agalactiae infection* and the adverse impacts of CPF on fish health, fish received *C. vulgaris* via their diets.

## Materials and methods

### Fish accommodation and experimental design

Healthy 360 Nile tilapia *(Oreochromis niloticus)*, weighing 35.6 ± 0.4 g, were obtained from the local freshwater fish farm and directly transported to the Kafrelsheikh provincial wet laboratory of Animal Health Research Institute. In the wet laboratory, fish were acclimatized for 14 days in experimental conditions: water temperature, pH, and salinity were 27.5 ± 0.5 °C, 7.9 ± 0.1, and 0.48 ± 0.1 g/L, respectively. Day after day, only one-third of tank water was exchanged with unchlorinated clean water to maintain suitable water parameters. Fish feed was offered twice a day at 0.9.30 a.m. and 03.00 p.m. at a rate of 5% fish of body weight, fish feed composition (Table 1): crude protein 30.2%, digestible energy 3450 kcal/kg as recommended for Nile tilapia [29].

**Table 1:** List of fish feed ingredients

Ingredient	%	Ingredient	%
Corn	24	MCP	1
Soya (44%)	33	Salt	0.15
Fish meal	21	Methionine	0.05
DDGs	4.5	Choline chloride	0.05
Corn gluten	15	Minerals premix	0.1
Soya oil	1	Vitamins premix	0.1

*Note* DDGs = Dried Distilled Grains

Fish were reared in water polluted with 10% of median lethal concentration (LC_50_) chlorpyrifos (CPF), a patent formulation manufactured by El Nasr Chemical Co., Egypt, 48% CPF, O, O-diethyl-O-(3,5,6-trichlor-2-pyridyl) phosphorothioate), for 2, 4, and 6 weeks. A trial to ameliorate CPF toxicity with dietary *Chlorella vulgaris*, which was provided the Faculty of Agriculture, Kafrelsheikh University. In four groups (G1–4) in triplicate, fish were randomly stocked into 12 glass aquaria (each aquarium measuring 80 × 40 × 40 cm, containing 30 fish). G1–2: Fish were fed on fed on *C. vulgaris* at a rate of 0 and 50 g/kg fish feed, respectively; the dose of *C. vulgaris* was recommended by Chen et al. [30] while G3–4 Fish were exposed to CPF (10% of LC_50_) and fed on *C. vulgaris* at a rate of 0 and 50 g/kg, respectively.

For sampling, fish was tranquilized by immersion in Tricaine methanesulfonate (MS-222) (SyncaineR, Syndel, Canada) at a dose of 40 mg/L water. Fish was euthanized by immersion in MS-222 solution of 250 mg/L water for 10 min following the methods described by Sherif et al. [31] and Eldessouki et al. [32].

### Median lethal concentration

To experiment with CPF stress, its LC_50_ was detected following the procedure developed by Reed and Muench [33]. Briefly, three hundred and thirty (330) Nile tilapia were equally stocked in 33 glass aquariums (40 × 40 × 50 cm), fish sub-divided into eleven groups (group = 3 aquaria), and every group was subjected to different concentrations of CPF (100, 90, 80, 70, 60, 50, 40, 30, 20, 10, and 0.0 µg/L). Mortalities were recorded for 72 h.

### Blood and serum analyses

Blood analyses of experimental Nile tilapia were performed using a hemocytometer and stain (Natt and Herrick) for red blood cell (RBC) and white blood cell (WBC) counts according to Stoskopf [34]. Hemoglobin content (Hb) was determined by the cyanmethemoglobin method [35] and packed cell volume (PCV) was measured using a centrifuge.

The serum of the experimental fish was examined for total protein (TP) [36], albumin (ALB), and globulin (GLO) [37]. Liver enzymes aspartate aminotransferase (AST) and alanine aminotransferase (ALT) [38] were colorimetrically measured using ELISA, and the ELISA-kit reagents were supplied by Diamond Diagnostic Co. (Holliston, USA).

### Gene expressions of antioxidant enzymes and immune

Gene expression in the head kidney of experimental Nile tilapia was performed for immunological cytokines interleukin (*IL) 1β, IL-10*, and tumor necrosis factor *(TNF-α)*, as well as antioxidant enzymes superoxide dismutase (SOD), glutathione peroxidase (GPx), and catalase (CAT). In Table 2, all primer sequences are present based on the National Center for Biotechnology Information (NCBI) database. All primers and kits were supplied by Sigma-Aldrich (Sigma-Aldrich Chemie GmbH, Steinheim, Germany). Quantitative polymerase chain reaction qPCR was carried out in a thermal cycler (AbiPrism 7300) (Applied Biosystems, USA). The quantitative fold alterations in the examined genes were calculated in relation to *β-*actin mRNA (household gene) by the 2^− DD^ CT method.

**Table 2:** Primers for cytokines, antioxidants enzymes, and household gene

Target gene	Primer sequence	Amplified segment Length	Annealing temperature	Accession number
*β-actin*	F: AGCAAGCAGGAGTACGATGAG R: TGTGTGGTGTGTGGTTGTTTTG	135 bp	58.5 °C 30 s	XM_003443127.5
*IL-1β*	F: T GCTGAGCACAGAATTCCAG R: GCTGTGGAGAAGAACCAAGC	172 bp	60 °C 30 s	XM_019365841.2
*IL-10*	F: CTGCTAGATCAGTCCGTCGAA R: GCAGAACCGTGTCCAGGTAA	94 bp	60 °C 30 s	XM_013269189.3
*TNF-α*	F: CCAGAAGCACTAAAGGCGAAGA R: CCTTGGCTTTGCTGCTGATC	82 bp	59.9 °C 30 s	AY428948.1
*SOD*	F: GGTGCCCTGGAGCCCTA R: ATGCGAAGTCTTCCACTGTC	377	56 °C 30 s	JF801727.1
*CAT*	F: TCCTGAATGAGGAGGAGCGA R: ATCTTAGATGAGGCGGTGATG	232	56 °C 30 s	JF801726.1
*GPx*	F: CCAAGAGAACTGCAAGAACGA R: CAGGACACGTCATTCCTACAC	237	58 °C 30 s	NM_001279711.1

*Note* interleukin (*IL)*, tumor necrosis factor *(TNF-α)*, superoxide dismutase *(SOD)*, glutathione peroxidase *(GPx)*, and catalase *(CAT)*

### Bacterial infection

At the end of each experimental period 2, 4, and 6 weeks, ten fish /group were injected intraperitoneally (IP) with pathogenic *S. agalactiae* with NCBI accession number (OL471408), and its median lethal dose (LD_50_) is 0.3 × 10^5^ CFU/ml [39]. In addition, pure saline solution (0.65%) was injected into ten fish as negative controls [40]. The injected fish were observed for 14 days to record the mortality rate (MR).

$$\text{M}\text{R}\left(\text{\%}\right) =\frac{\text{n}\text{u}\text{m}\text{b}\text{e}\text{r} \,\text{o}\text{f} \,\text{d}\text{e}\text{a}\text{t}\text{h}\text{s} \,\text{i}\text{n} \,\text{a} \,\text{s}\text{p}\text{e}\text{c}\text{i}\text{f}\text{i}\text{c} \,\text{p}\text{e}\text{r}\text{i}\text{o}\text{d} }{\text{t}\text{o}\text{t}\text{a}\text{l} \,\text{p}\text{o}\text{p}\text{u}\text{l}\text{a}\text{t}\text{i}\text{o}\text{n} \,\text{d}\text{u}\text{r}\text{i}\text{n}\text{g} \,\text{t}\text{h}\text{a}\text{t} \,\text{p}\text{e}\text{r}\text{i}\text{o}\text{d}}\times 100$$

Whereas the relative protection level (RLP) was verified among the challenged fish following the equation [41]:

$$\text{R}\text{L}\text{P}\text{\%}=(1-\frac{\text{\%}\text{d}\text{e}\text{a}\text{t}\text{h}\text{s} \,\text{i}\text{n} \,\text{t}\text{h}\text{e} \,\text{t}\text{r}\text{e}\text{a}\text{t}\text{e}\text{d} \,\text{g}\text{r}\text{o}\text{u}\text{p}}{\text{\%}\text{d}\text{e}\text{a}\text{t}\text{h}\text{s} \,\text{i}\text{n} \,\text{t}\text{h}\text{e} \,\text{c}\text{o}\text{n}\text{t}\text{r}\text{o}\text{l} \,\text{g}\text{r}\text{o}\text{u}\text{p}})\times 100$$

### Biosafety protocol

The experiment procedure followed the biosafety measures on the pathogen safety data sheets (Infectious substances–*S. agalactiae*, Pathogen Regulation Directorate [42].

### Statistical examination

The impacts of CPF and *C. vulgaris* algae were statistically assessed on different health parameters of the experimental Nile tilapia. The mean and standard error of the collected values were obtained with the ANOVA test and Duncan’s Multiple Range using SPSS software version 22. Statistical significances were considered at P-values ≤ 0.05.

## Results

### Median lethal concentration (LC_50_)

The LC_50_-_72 h_ was detected to be 85.8 µg /L for experimental Nile tilapia with body weight 35.6 ± 0.5 g at water temperature, pH, and salinity of 27.5 ± 0.5 °C, 7.9 ± 0.1, and 0.48 ± 0.1 g/L, respectively.

### Immunological and antioxidants responses

**Fig. 1:**
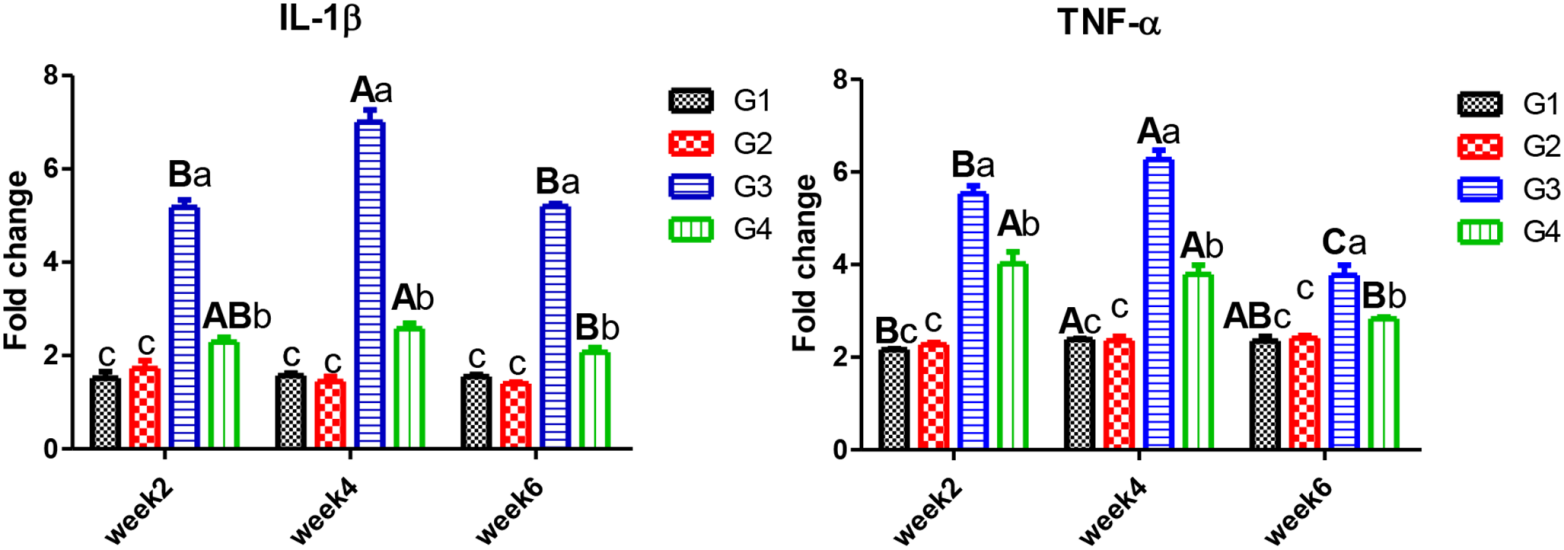
Gene expression of *IL-1β* and *TNF-α* in the head kidney of Nile tilapia. *Note* W2; week2, W4; week4, W6; week6. G1; control, G2; fish fed 50 g C. vulgaris /kg fish feed, G3; fish exposed to CPF (10% of LC50), G4; Fish exposed to CPF (10% of LC50) and fed 50 g *C. vulgaris* /kg fish feed. Different capital letters indicate significant difference based on the time factor in the same group, different small letters indicate significant difference based on the treatment factor within the same period at *P* ≤ 0.05

**Fig. 2:**
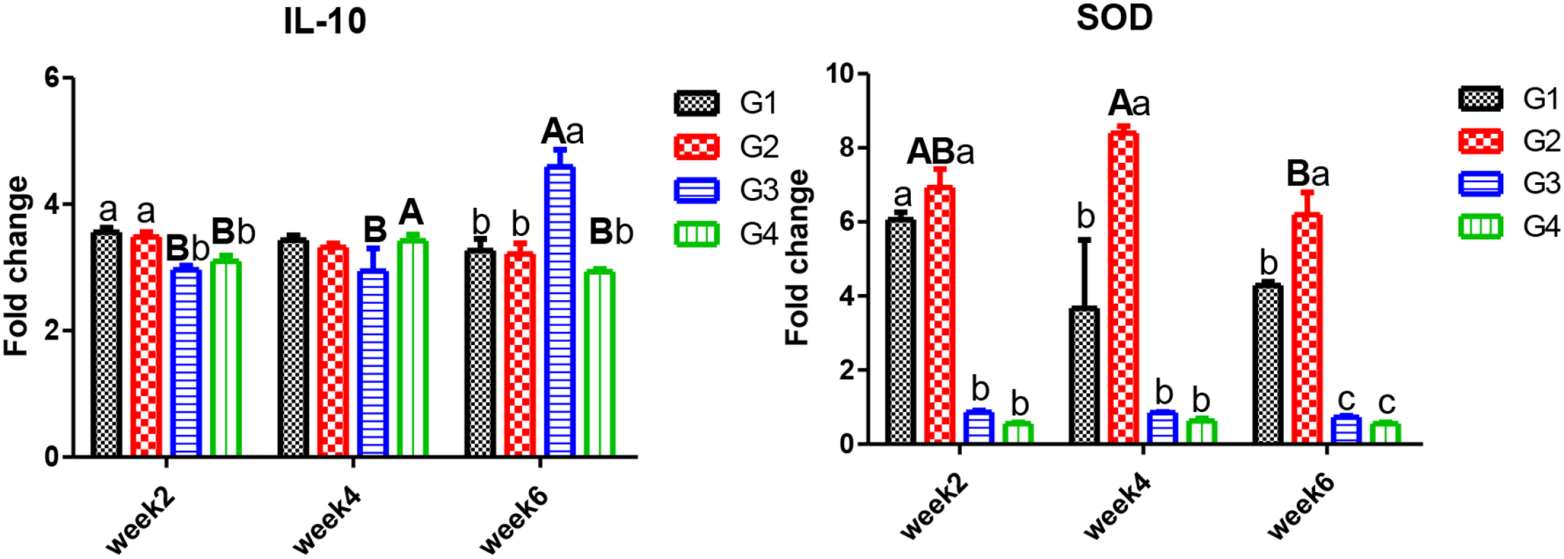
Gene expression of *IL-10* and *SOD* in the head kidney of Nile tilapia. *Note* W2; week2, W4; week4, W6; week6. G1; control, G2; fish fed 50 g C. vulgaris /kg fish feed, G3; fish exposed to CPF (10% of LC50), G4; Fish exposed to CPF (10% of LC50) and fed 50 g *C. vulgaris* /kg fish feed. Different capital letters indicate significant difference based on the time factor in the same group, different small letters indicate significant difference based on the treatment factor within the same period at *P* ≤ 0.05

In Figs. 1 and 2, some cytokines *IL-1β, TNF-α*, and *IL-10* gene expression were determined in the head kidney to assess CPF impacts on Nile tilapia’s immunity. Time trend, exposure to CPF (G3) resulted in pro-inflammatory *IL-1β* and *TNF-α* upsurge in four weeks after that declined after six weeks. There were no significant alterations in either controls or those who received *C. vulgaris.* After 2, 4, and 6 weeks of exposure, *C. vulgaris* supplementation *decreased IL-1β*, and *TNF-α* remained significantly higher than the control. The anti-inflammatory *IL-10* gene expression had an opposite trend with pro-inflammatory *IL-1β* and *TNF-α.*

### Antioxidants gene expression

**Fig. 3:**
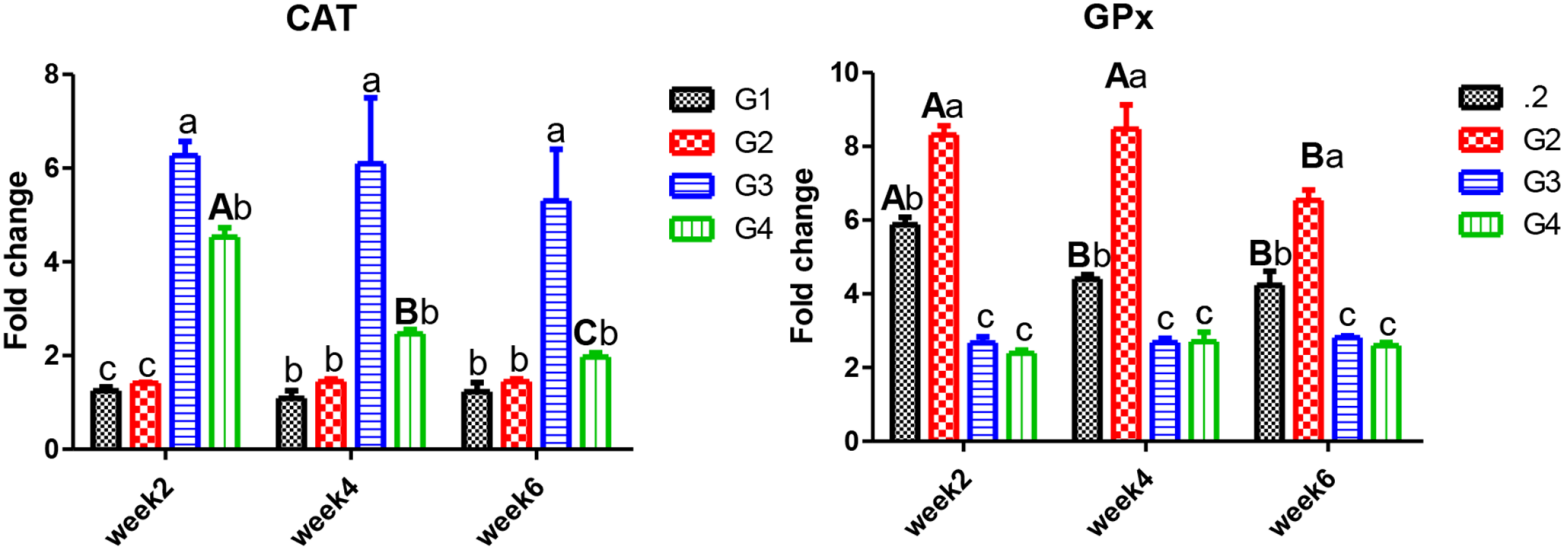
Gene expression of *CAT* and *GPx* in the head kidney of Nile tilapia. *Note* W2; week2, W4; week4, W6; week6. G1; control, G2; fish fed 50 g C. vulgaris /kg fish feed, G3; fish exposed to CPF (10% of LC50), G4; Fish exposed to CPF (10% of LC50) and fed 50 g *C. vulgaris* /kg fish feed. Different capital letters indicate significant difference based on the time factor in the same group, different small letters indicate significant difference based on the treatment factor within the same period at *P* ≤ 0.05

Antioxidants enzymes, one of the defense mechanisms to combat the contaminants, SOD (Fig. 2) and GPx (Fig. 3), showed insignificant differences in the CPF and CPF plus *C. vulgaris* groups. In contrast, those exposed to CPF significantly decreased with time, and the supplementation with *C. vulgaris* significantly enhanced antioxidant status. With time, the antioxidant responses to CPF exposure remained low compared to the other groups. While gene expression of CAT (Fig. 3) was significantly increased with exposure and with time, it was also noted that *C. vulgaris* could reduce such an upsurge.

### Blood analyses in Nile tilapia

In Table 3, blood indices confirmed the deleterious impacts of CPF exposure; values of RBCs, Hb, and PCV indicated Nile tilapia was anemic after six weeks of exposure (G3), and fish could restore normal values after six weeks of *C. vulgaris* supplementation (G4). On the contrary, WBCs were significantly increased on CPF exposure.

In Table 3, after weeks of CPF exposure, serum TP, GLO, and ALB significantly decreased with time trend. After four weeks, fish exposed to CPF suffered from low TP and GLO with an insignificant decrease of ALB, whereas, after six weeks, all serum proteins declined, including ALB value.

**Table 3:** Blood and serum analyses of the experimental Nile tilapia

Item	RBC×10^6^				WBCs ×10^3^				Hb g/dl			
	G1	G2	G3	G4	G1	G2	G3	G4	G1	G2	G3	G4
W2	2.43 ± 0.05^ab^	2.5 ± 0.03^a^	2.21 ± 0.12^Ab^	2.34 ± 0.02^ab^	59.57 ± 0.4^c^	58.57 ± 1.47^c^	70 ± 1.32^a^	64.03 ± 0.87^b^	8.23 ± 0.18^ab^	8.51 ± 0.11^a^	7.51 ± 0.4^Ab^	7.94 ± 0.08^ab^
W4	2.62 ± 0.02^a^	2.7 ± 0.04^a^	1.96 ± 0.08^Ac^	2.28 ± 0.14^b^	59.7 ± 1.06^b^	58.4 ± 1.25^b^	68.1 ± 1.35^a^	63 ± 1.71^b^	8.89 ± 0.08^a^	9.17 ± 0.15^a^	6.65 ± 0.27^Ac^	7.76 ± 0.46^b^
W6	2.45 ± 0.13^a^	2.57 ± 0.14^a^	1.61 ± 0.02^Bb^	2.27 ± 0.04^a^	57.7 ± 1.2^c^	56 ± 1.2^c^	66.7 ± 0.5^a^	62.7 ± 1.24^b^	8.32 ± 0.46^a^	8.74 ± 0.47^a^	5.46 ± 0.08^Bb^	7.71 ± 0.12^a^
	PCV %				TP g/dL				GLO g/dL			
W2	25.87 ± 0.54^ab^	26.65 ± 0.38a	23.55 ± 1.23^Ab^	24.9 ± 0.26^ab^	5.93 ± 0.27	5.67 ± 0.19^B^	5 ± 0.42^A^	5.2 ± 0.3^A^	2.78 ± 0.25	2.51 ± 0.16^B^	1.99 ± 0.4	2.05 ± 0.33^A^
W4	27.88 ± 0.25^a^	28.75 ± 0.47a	20.86 ± 0.87^Ac^	24.3 ± 1.45^b^	5.55 ± 0.18^b^	6.26 ± 0.03^Aa^	4.33 ± 0.07^ABd^	4.83 ± 0.04^Ac^	2.45 ± 0.16^b^	3.17 ± 0.07^Aa^	1.21 ± 0.06^d^	1.73 ± 0.02^ABc^
W6	26.12 ± 1.43^a^	27.4a ± 1.44	17.11 ± 0.26^Bb^	24.2 ± 0.4^a^	5.9 ± 0.06^b^	6.38 ± 0.08^Aa^	3.6 ± 0.15^Bd^	4.18 ± 0.09^Bc^	2.83 ± 0.09^b^	3.33 ± 0.08^Aa^	1.32 ± 0.07^c^	1.08 ± 0.06^Bc^
	ALB g/dL				ALT IU/L				AST IU/L			
W2	3.15 ± 0.03	3.16 ± 0.12	3.01 ± 0.03^A^	3.15 ± 0.03	27.4 ± 0.47^c^	28.23 ± 0.5^Ac^	42.5 ± 0.76^Ba^	39.2 ± 0.6^Ab^	31.5 ± 0.03	31.6 ± 0.12	30.1 ± 0.03^A^	31.5 ± 0.03
W4	3.1 ± 0.05	3.1 ± 0.04	3.12 ± 0.1^A^	3.1 ± 0.03	28.3 ± 0.4^c^	26.6 ± 0.66^ABc^	53.3 ± 5.05^Aa^	39 ± 1.1^Ab^	31 ± 0.05	31. ± 0.04	31.2 ± 0.1^A^	31 ± 0.03
W6	3.07 ± 0.04^a^	3.05 ± 0.04^a^	2.28 ± 0.1^Bb^	3.09 ± 0.06^a^	27.8 ± 0.54^c^	25.1 ± 0.32^Bd^	61.7 ± 1^Aa^	34.3 ± 0.67^Bb^	30.7 ± 0.04^a^	30.5 ± 0.04^a^	22.8 ± 0.1^Bb^	30.9 ± 0.06^a^

*Note* W2; week2, W4; week4, W6; week6. G1; control, G2; fish fed 50 g C. vulgaris /kg fish feed, G3; fish exposed to CPF (10% of LC50), G4; Fish exposed to CPF (10% of LC50) and fed 50 g *C. vulgaris* /kg fish feed. Different capital letters indicate significant difference based on the time factor in the same group, different small letters indicate significant difference based on the treatment factor within the same period at *P* ≤ 0.05

### Liver enzymes

In Table 3, liver enzymes ALT (42.5, 53.3, and 61.7 IU/L) and AST (30.1, 31.2, and 22.8) significantly increased after CPF exposure and increased with time, while the *C. vulgaris* supplementation could partially restore normal status as it was still higher than un-exposed fish ALT (39.2, 39, and 34.3 IU/L) and AST (31.5, 31, and 30.9 IU/L) in week 2, week 4, and week 6, respectively.

### Bacterial infection with *S. agalactiae*

**Fig. 4:**
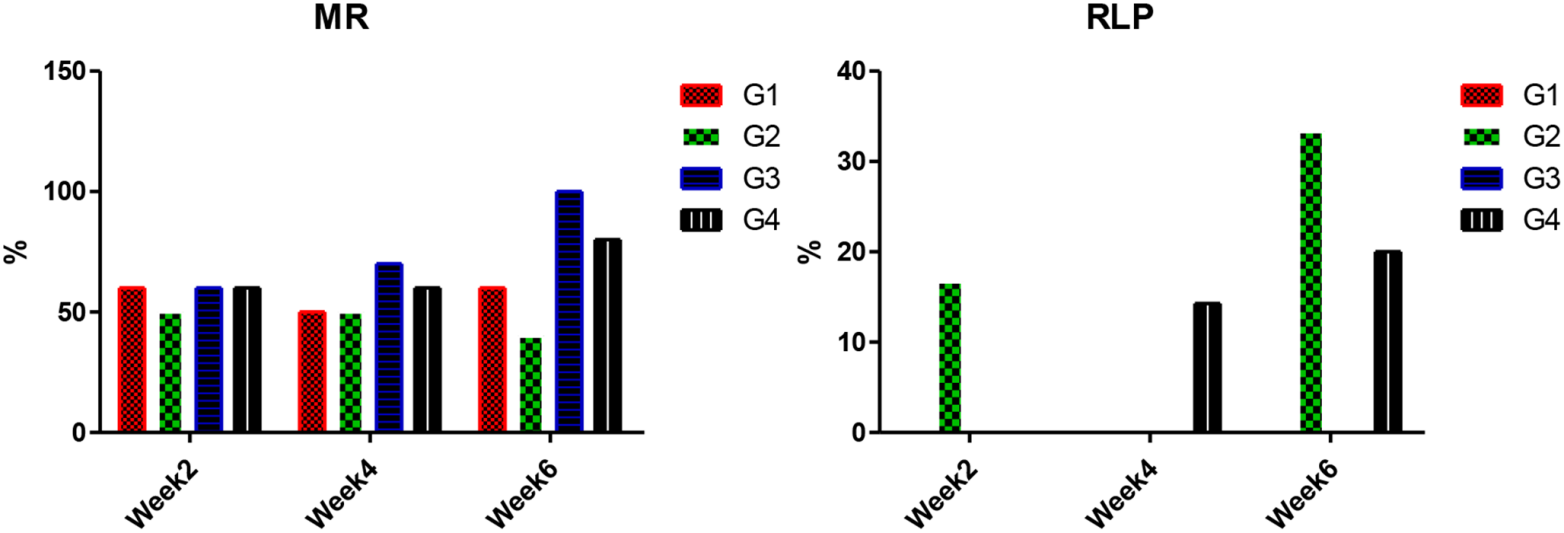
Mortality rate (MR) and relative level of protection (RPL) of Nile tilapia challenged with*S. agalactiae.*(*n* = 10). Note: W2; week2, W4; week4, W6; week6. G1; control, G2; fish fed 50 g C. vulgaris /kg fish feed, G3; fish exposed to CPF (10% of LC50), G4; Fish exposed to CPF (10% of LC50) and fed 50 g *C. vulgaris* /kg fish feed. Different capital letters indicate significant difference based on the time factor in the same group, different small letters indicate significant difference based on the treatment factor within the same period at *P* ≤ 0.05

In Fig. 4, a low MR was recorded in Nile tilapia challenged with LD_50_ of *S. agalactiae* fed on *a C. vulgaris* supplemented diet 50, 50, and 40, compared to the control fish; 60, 50, and 60% in week 2, week 4, and week 6, respectively, giving an RPL of 16.67% (week2), 0 (week4), and 33.33% (week6). Nile tilapia exposed to CPF and fed on dietary *C. vulgaris (G4)* showed a decrease in MR 60%, 60, and 80%compared with those exposed to CPF (G3) 60%, 60%, 100% in week 2, week 4, and week 6, respectively. Dietary *C. vulgaris* provided a RPL of 0, 14.29, and 20% against CPF pollution for week 2, week 4, and week 6, respectively, and challenged with *S. agalactiae*.

**Fig. 5:**
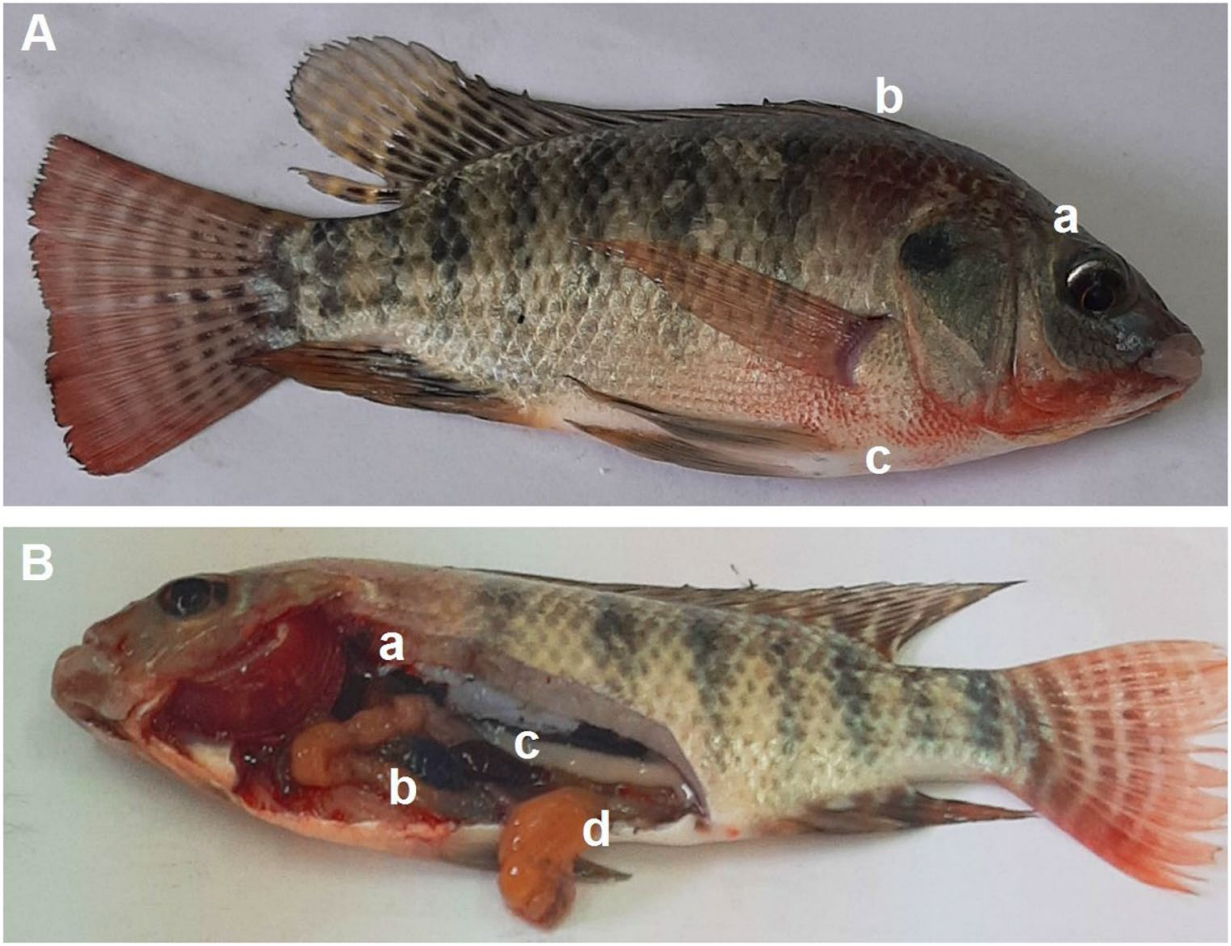
Nile tilapia (control group) infected with *S. agalactiae.*(**A**) Fish showed exophthalmia (**a**), skin hemorrhages dorsal (**b**) and ventral abdomen extended to mouth (**c**). (**B**) Fish showed dark liver (**a**), distended gall bladder with greenish content (**b**), splenomegaly (**c**), and partially empty intestine (**d**)

In Fig. 5 (A), Nile tilapia (G1) of the control group challenged with S. agalactiae, showed slight exophthalmia and skin hemorrhages (b, c). Post-mortem changes (B) were an empty intestinal tract, dark liver (a), distended gallbladder (b), splenomegaly (c), and partially empty intestine (d).

**Fig. 6:**
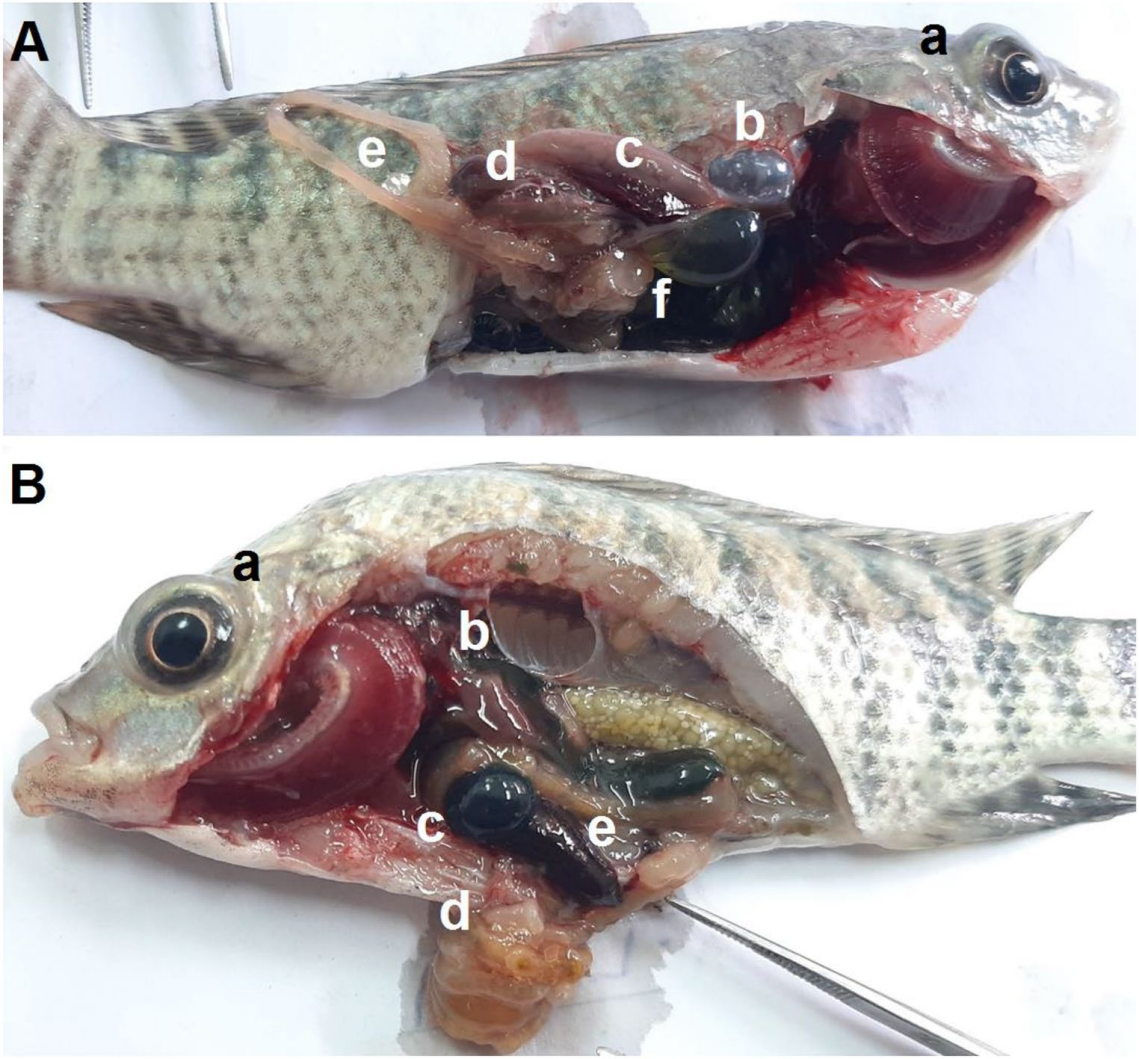
Nile tilapia infected with *S. agalactiae.* (**A**) Fish exposed to CPF, exophthalmia (**a**), turbid air bladder (**b**), splenomegaly (**c**), friable liver (**d**), empty intestinal tract (**e**), distended gall bladder with light yellow-greenish content (**f**). (**B**) Fish exposed to CPF and fed on dietary *C. vulgaris*, exophthalmia (**a**), dark brownish liver (**b**), distended gall bladder with dark greenish content (**c**), empty clumped intestinal tract (**d**), and splenomegaly (**e**)

In Fig. 6 (A), CPF-exposed fish (G3) suffered from exophthalmia (a), friable liver (d), distended gallbladder with clear content (f), and splenomegaly (c), and empty intestinal tract (e).

In Fig. 6 (B), fish exposed to CPF and received dietary C. vulgaris (G4) showed exophthalmia, dark-brownish liver, distended gallbladder with dark greenish content, splenomegaly, and empty clumped intestinal tract.

## Discussion

In this study, the LC_50_-_72 h_ of CPF was detected to be 85.8 µg /L for experimental Nile tilapia. Near to our findings, LC_50_-_48 h_ was 90 µg/L of CPF for Nile tilapia [43] and LC_50_-_96 h_ 105.3 µg/L ten spotted live-bearer *(Cnesterodon decemmaculatus)* [44]. The higher values recorded for the rohu *(Labeo rohita)* LC_50_-_96 h_ were 442.8 µg CPF /L [45]. Whereas the lower values of LC_50_-_48 h_ were 2.26 µg CPF /L for Pejerrey *(Odontesthes bonariensis)* [46] and 5.47 µg /L for the larvae of banded gourami *(Trichogaster fasciata)* [47]. These differences in LC_50_ value could be attributed to fish spp. as weight and physiological status or water conditions as temperature, salinity, and pH.

Sub-lethal concentrations of some contaminants drastically impact the immunity of aquatic animals, as many researchers found that gene expression of cytokines *(IL-1β, IL-8*, and *TNF-α)* was significantly altered [8, 48, 49].

In this study, exposure to 10% of CPF LC_50_ resulted in pro-inflammatory *IL-1β* and TNF-α upsurge in four weeks, while they declined after six weeks. The anti-inflammatory *IL-10* gene expression had an opposite trend with pro-inflammatory. Similarly, gene expression of *IL-1β* was upsurged in the head kidney of Chinook salmon reared in water polluted with CPF at 7.3 µg/L [50]. In addition, common carp *(Cyprinus carpio)* exhibited similar responses of *IL-1β* expression at 1.16 and 11.6 µg/L of CPF for 40 days with declining after 20 days recovery period [51], with the same concentration and period *TNF- α, IL-6*, and *IL-8* in both head kidney and spleen of common carp showed similar findings [52]. From our results, dietary *C. vulgaris* enhanced fish health by decreasing the exaggerated immune responses of *IL-1β* to CPF stress, whereas *TNF-α* remained significantly higher than the control. In consistence, CPF-induced toxicity in common carp resulted in high *IL-10* and *TNF-α* expression genes that could be mitigated by using both dietary *C. vulgaris* [52]. Different findings, 10% dietary *C. vulgaris* to fish diets significantly increased the expression of splenic and hepatic *IL-1β* and *TNF-α* in Nile tilapia exposed to deltamethrin toxicity [53]. This difference could be due to the high concentration of the organophosphorus compound.

The experimental Nile tilapia exposed to CPF showed a decline in SOD and GPx gene expression in the head kidney, while CAT increased after six weeks of exposure. Similarly, SOD activity decreased in the freshwater gastropod *(Bellamya bengalensis)* on the 20th day, with a significant increase in CAT under the interactions of elevated water temperature and CPF contamination [54]. In addition, SOD activity was reduced in common carp exposed to CPF for 40 days in the spleen and head kidney at 1.16 and 11.6 µg/L, followed by an increase after 20 days of recovery [55]. In consistency with our findings, exposure of zebrafish *(Danio rerio)* to CPF resulted in the downregulation of SOD and GPx activities but with a decrease in CAT activity [56, 57]. These findings could be explained by the fact that the interaction of GPx with electrophilic compounds can inhibit its activity [58], such as the metabolite of CPF in zebrafish [59].

Antioxidant activities of SOD and CAT enzymes in living organisms could eliminate and neutralize the reactive oxygen species (ROS) generated in response to exposure to toxic compounds [60, 61]. In this work, *C. vulgaris* significantly enhances SOD and GPx gene expression and could reduce such an upsurge of CAT gene expression, counteracting the CPF impacts. These findings may be due to the composition of *C. vulgaris*, which contains flavonoids, tocopherols, chlorophyll, carotenoids, and polyphenols, which could combat generated oxidative stress resulting from exposure to streptozotocin [62] and penoxsulam herbicide [23] also, it contains β-glucan, which can induce growth and antioxidant enzymes in several aquatic animals [63].

In our findings, blood indices such as RBCs, Hb, and PCV indicated an anemic status of Nile tilapia after only two weeks of CPF exposure. On CPF exposure, a significant reduction in RBCs, Hb, and PCV values in Nile tilapia [64], Caspian brown trout *(Salmo trutta caspius)* at a concentration of 26 µg/L for 20 days [65]. Different explanations for previous findings include the increases in the rate of RBC damage or inhibition of its formation, which could be due to a decline of serum iron concentrations, thereby lowering Hb synthesis in common carp exposed to CPF [66], Mozambique tilapia *(Oreochromis mossambicus)* exposed to cadmium and CPF [67]. Another explanation is that RBC became more fragile on CPF exposure because of the generated oxidative stress that impacted erythrocyte membranes [68]. In contrast, Jaffer et al. [69] claimed that common carp exposed to CPF (52, 79, and 158 µg/L) did not alter after three successive weeks of exposure. Blood analyses of the experimental Nile tilapia revealed a significant increase in WBCs after CPF exposure. Similarly, WBCs, neutrophils, and lymphocytes were increased in fish exposed to different contaminants, such as pesticides, to cope with the generated immunosuppression status [70].

In this work, CPF exposure resulted in a decline of serum TP, GLO, and ALB. Similarly, exposure to CPF decreased the TP and GLO values in common carp [5]. Other reports supported these findings, and they mentioned that CPF could stimulate significant alterations in blood biochemical indices that could result in immune-compromised status in fish [5, 43]. Fish could restore normal blood indices values after six weeks of receiving dietary C. *vulgaris*. Similarly, Sayed et al. [21] reported that dietary C. *vulgaris* at a rate of 5% of fish feed for 15 days could enhance the serum total protein, globulin, and albumin of s in African sharp-tooth catfish impacted by the toxicity microplastics (500 mg/kg fish feed). Also, Galal et al. [23] stated that *C. vulgaris* could protect fish health by maintaining normal blood parameters of Nile tilapia exposed to sub-lethal concentrations of penoxsulam (herbicide).

The liver is the primary organ for detoxification and processing of toxicants, becoming the site of bioaccumulation, for example, CPF toxicity in Indian carp *(Catla catla), Labeo rohita, and Cirrhinus mrigala* [71]. In this work, liver enzymes ALT and AST significantly increased after CPF exposure. Similarly, an upsurge in serum AST and ALT values was found in Nile tilapia exposed to CPF acute toxicity [72], golden mahseer (*Tor putitora)* [73], and freshwater crayfish (*Pontastacus leptodactylus)* [74]. However, dietary C. *vulgaris* could partially decrease the serum liver enzymes of the experimental Nile tilapia; they were still higher than in the control ones (unexposed to CPF). Similar results were obtained with Nile tilapia exposed to diazinon-toxicity and received dietary *Spirulina*, *Chlorella*, or their mixture [75].

In this work, after the *S. agalactiae* challenge, Nile tilapia exposed to CPF exhibited higher MR than other groups and reached 100% after six weeks of exposure. Similarly, common carp exposed to pesticides (CPF) were vulnerable to infectious pathogens as they were stressed and immunosuppressed [6, 76]. Meanwhile, dietary C. *vulgaris* could lower MR% and raise the RPL%. Similarly, *C. vulgaris* has antibacterial properties against many Gram-positive and G-negative bacteria [77]. The decline of MR and high RLP could be anticipated after the previously mentioned results concerning the enhancements of immune-oxidative gene expression and blood analyses of Nile tilapia received dietary *C. vulgaris*. These findings could be due to bioactive compounds secreted by microalgae preventing microbial growth [20–23].

Experimental Nile tilapia, challenged against *S. agalactiae*, harbored signs of septicemic diseases such as skin hemorrhages and exophthalmia, whereas post-mortem lesions were empty intestinal tract, distended gallbladder, and splenomegaly. Similarly, Nile tilapia infected with *S. agalactiae* had signs of bacterial septicemia such as exophthalmia, splenomegaly, hepatomegaly, and distended gall bladder [37, 78].

## Conclusion

CPF stress could be evaluated in Nile tilapia by measuring gene expression of cytokines and antioxidant enzymes. Even *C. vulgaris* at a dose of 50 g/kg fish feed could ameliorate immunosuppression and oxidative impacts, but it could not fully help fish restore normal health parameters. The anemic status of Nile tilapia was the most prominent sign of stressed fish. Nile tilapia exposed to CPF and challenged against *S. agalactiae* had the highest mortalities compared to the control.

## Data Availability

Data is available on request from the corresponding author.

## Code availability

Code availability Not applicable.
